# Macrophage activation syndrome in adults: Characteristics, outcomes, and therapeutic effectiveness of etoposide-based regimen

**DOI:** 10.3389/fimmu.2022.955523

**Published:** 2022-09-15

**Authors:** Lingbo He, Shuyan Yao, Ruoxi Zhang, Menghan Liu, Zhengjie Hua, Heshan Zou, Zhao Wang, Yini Wang

**Affiliations:** ^1^ Department of Hematology, Beijing Friendship Hospital, Capital Medical University, Beijing, China; ^2^ Department of General Practice, Beijing Friendship Hospital, Capital Medical University, Beijing, China

**Keywords:** hemophagocytic lymphohistiocytosis (HLH), macrophage activation syndrome (MAS), autoimmune disease (AID), cytokine, etoposide, adult

## Abstract

**Objectives:**

To describe the clinical characteristics and outcomes of adult macrophage activation syndrome (MAS) patients and to provide experience for the treatment.

**Methods:**

Adult patients with MAS admitted to Beijing Friendship Hospital from December 2014 to September 2021 were enrolled in this study. Clinical data of patients were collected and analyzed.

**Results:**

A total of 118 adult MAS patients entered this study. MAS was the first manifestation in 43 (36.4%) patients, while 75 (63.6%) developed MAS after the diagnosis of autoimmune disease (AID) with a median diagnostic interval of 2 (0.5–359) months. Eighty-two patients were initially treated with glucocorticoid-based regimen; the overall response (OR) rate at the 2-week posttreatment was 37.8%. Forty-five patients switched to etoposide-based regimen, and the OR rate was 84.4%. Thirty-six patients were initially treated with etoposide-based regimen, and the OR rate at the 2-week posttreatment was 80.6%. Serum IL-18 (P = 0.021), IFN-γ (P = 0.013), IP-10 (P = 0.001), IL-10 (P = 0.041), IL-1RA (P < 0.001), and TNF-α (P = 0.020) levels of patients were significantly decreased in the remission phase than in the active phase. Levels of SDF-1α (P = 0.018) and IL-7 (P = 0.022) were higher in refractory patients, while the GRO-α level had a strong tendency toward statistical significance (P = 0.050). The probability of overall survival (OS) at 3, 6, and 36 months after HLH diagnosis were 89.8%, 89.0%, and 87.9%, retrospectively. The active MAS status at the 2-week post initial treatment [P = 0.009, HR = 15.281, 95% CI, (0.1.972, 118.430)] and baseline neutrophil count (Neu) <1.5 × 10^9^/l [P = 0.017, HR = 3.678, 95% CI, (1.267, 10.672)] were negative prognostic factors.

**Conclusion:**

MAS typically occurs within 2 months after the onset of autoimmune disease in adults. SDF-1α, IL-7, and GRO-α could be used to predict refractory MAS. The etoposide-based regimen is effective and tolerable for adult MAS.

## Introduction

Macrophage activation syndrome (MAS) is a potentially life-threatening complication of rheumatic diseases, which was regarded as a subtype of secondary hemophagocytic lymphohistiocytosis (HLH) (1). It is most prevalent and well described in systemic-onset juvenile idiopathic arthritis (sJIA) patients (2, 3). To date, MAS has been reported in various autoimmune diseases such as systemic lupus erythematosus (SLE), adult-onset Still’s disease (AOSD), and Kawasaki disease (4, 5). Cardinal symptoms and laboratory abnormalities include prolonged fever, hepatosplenomegaly, pancytopenia, and elevated levels of serum ferritin (SF) (6). The overlap in clinical manifestations between MAS and acute exacerbation of autoimmune disease (AID) challenges the early identification of MAS (7). Currently, there are no validated management guidelines for MAS due to the lack of randomized control trials. According to pediatric experiences, glucocorticoid pulse therapy is the prevailing treatment for MAS, but approximately 50% of adult patients are unresponsive (8–10). Thus, it is essential to improve the understanding of MAS for early diagnosis and appropriate treatment, especially in adult patients. However, data on adult patients are rather limited (11). In this study, we aimed to give a description of the clinical characteristics and prognosis of adult MAS and to provide our experience of treatment strategies.

## Patients and methods

### Patients

Patients were consecutively enrolled in this study from December 2014 to September 2021 at Beijing Friendship Hospital, Capital Medical University. The inclusion criteria of this study were as follows: (1) meeting the HLH-2004 diagnostic criteria; (2) age over 18 years; (3) diagnosed with AID. The excluding criterion was identification of evidence for other subtypes of HLH.

### Treatment

The glucocorticoid-based regimen consisted of the following: methylprednisolone 500–1,000 mg for 3 consecutive days, alone or combined with immunosuppressants (cyclosporine A, hydroxychloroquine, methotrexate, cyclophosphamide, etc.) or intravenous immunoglobulin (IVIG). The dose of glucocorticoid was then reduced depending on the underlying autoimmune disease.

The etoposide (VP-16)-based regimen consisted of the following: HLH-94, HLH-2004, or the modified doxorubicin-etoposide-methylprednisolone (DEP) regimen (12). The dose of etoposide was determined according to the treatment plan, such as 150 mg/m^2^ for the HLH-94/2004 regimen and 100 mg/m^2^ for the DEP regimen.

### Assessment of therapy

*Evaluation indicators:* The quantifiable symptoms and laboratory markers of HLH were evaluated every 2 weeks before HSCT, including levels of soluble CD25 (sCD25), SF, and triglyceride (TG); hemoglobin (Hgb); neutrophil counts (Neu); platelet counts (PLT); alanine aminotransferase (ALT); CNS involvement; and presence of hemophagocytosis in pathology specimens.

*Complete response (CR):* It was defined as normalization of all the above quantifiable symptoms and laboratory markers of HLH.

*Partial response (PR):* It was defined as at least a 25% improvement in two or more quantifiable symptoms and laboratory markers compared with the baseline. The specific markers should meet the criteria as follows: sCD25 response was 1.5-fold decreased; SF and TG decreased by at least 25%; for patients with an initial Neu of <0.5 × 10^9^/l, a response was defined as an increase by at least 100% to >0.5 × 10^9^/l; for patients with a Neu of 0.5 to 2.0 × 10^9^/l, an increase by at least 100% to >2.0 × 10^9^/l was considered a response; and for patients with ALT >400 U/l, response was defined as an ALT decrease of at least 50%.

*No response (NR):* Patients who did not meet any response criteria were defined as “no response.”

### Refractory/relapsed MAS

Refractory MAS was defined as treatment with glucocorticoid pulse therapy (methylprednisolone 500-1000mg/day for 3 consecutive days) without achieving at least PR. Relapsed MAS was defined as meeting at least three HLH-2004 diagnostic criteria after achieving CR.

### Measurement of serum cytokine levels

Serum cytokine levels were measured using multiplex bead array assay, including MIP-1α, SDF-1α, IL-27, IL-1β, IL-2, IL-4, IL-5, IP-10, IL-6, IL-7, IL-8, IL-10, eotaxin, IL-12p70, IL-13, IL-17A, IL-31, IL-1RA, RANTES, IFN-γ, GM-SCF, TNF-α, MIP-1β, IFN-α, MCP-1, IL-9, TNF-β, GROα, IL-1α, IL-23, IL-15, IL-18, IL-21, and IL-22.

### Survival and follow-up

All patients were followed until 31 December 2021, or the date of death. The overall survival was calculated from the date of MAS diagnosis to the last follow-up or the death from any cause.

### Statistical analysis

SPSS 26.0 statistical software was adopted. All data that were not distributed normally were represented by median and range, and comparisons of multiple samples between groups were performed by the Wilcoxon rank-sum test. The chi-squared test was used for the comparison of proportions. Overall survival probabilities were estimated by the Kaplan–Meier method, and the log-rank test was used to assess differences in univariate analysis. Multivariate analysis was performed using Cox proportional hazards regression. In all analyses, P < 0.05 indicated statistical significance.

## Results

### Clinical and laboratory findings at diagnosis

A total of 118 patients were eligible for inclusion in the analysis, including 26 (26/118, 22.0%) men and 92 (92/118, 78.0%) women. The ratio of men to women was 1: 3.5, and the median age was 31 (18–76) years. Clinical and laboratory findings of patients are summarized in **Table 1**. Fever (118/118, 100%) was the most prominent manifestation. The most remarkable laboratory feature was hyperferritinemia, with 49 patients (49/118, 41.5%) presenting with SF >6,000 µg/l. The proportion of patients with Neu <1.0 × 10^9^/l, Hgb <90 g/l, and PLT <100 × 10^9^/l was 17.8% (21/118), 39.8% (47/118), and 56.8% (49/118), retrospectively. The sCD25 level was high in 73.8% (59/80) of patients. Low NK cell activity was observed in 69.7% (46/66) of patients. Concomitant infections were identified in 45 (45/118, 39.0%) patients. Viruses (38/118, 32.2%) were the most frequent infectious organisms, including EBV [serum: 9.7 × 10^2^ (0–6.5 × 10^4^) copies/ml; peripheral blood mononuclear cells: 1.45 × 10^3^ (1.3 × 10^3^–1.6 × 10^3^) copies/ml], CMV (1.1 × 10^4^ (4.2 × 10^2^–8.9 × 10^4^) copies/ml), HHV-6, HHV-7, HSV, Parvovirus B19, and Coxsackie B5. Seven patients (7/118, 5.9%) had fungal infections, including *Candida albicans* and *Candida glabrata*. Six patients (6/118, 5.1%) had bacterial infections, including *Pseudomonas aeruginosa*, *Klebsiella pneumoniae*, *Citrobacter brucella*, *Escherichia coli*, *Listeria*, *Staphylococcus*, and *Enterococcus faecium.* Two patients (2/118, 1.7%) had mycoplasma pneumoniae infection.

**Table 1:** Baseline demographic characteristics, clinical manifestations, and laboratory features of patients.

	All patients (n = 118)	Group 1 (n = 82)^a^	Group 2 (n = 36)^b^	P value
Demographics				
Gender, n (%)				
Male	26 (22.0%)	18 (22.0%)	8 (22.2%)	0.974
Female	92 (78.0%)	64 (78.0%)	28 (77.8%)	
Age, years, media (range)	31 (18-76)	33 (18-76)	29 (18-72)	0.065
Underlying autoimmune disease, n (%)				
AOSD	64 (54.2%)	46 (56.1%)	18 (50.0%)	–
SLE	22 (18.6)	16 (19.5%)	6 (16.7%)	
UCTD	19 (16.1%)	12 (14.6%)	7 (19.4%)	
RA	5 (4.2%)	4 (4.9%)	1 (2.8%)	
DM	3 (2.5%)	1 (1.2%)	2 (5.6%)	
SS	2 (1.7%)	1 (1.2%)	1 (2.8%)	
AIH	2 (1.7%)	2 (2.4%)	0 (0.0%)	
BD	1 (0.8%)	0 (0.0%)	1 (2.8%)	
Clinical manifestations, n (%)				
Fever	118 (100.0%)	82 (100.0%)	36 (100.0%)	–
Skin rashes	66 (55.9%)	46 (56.1%)	20 (55.6%)	0.956
Joint pain	63 (53.4%)	44 (53.7%)	19 (52.8%)	0.930
Hepatomegaly	11 (9.3%)	8 (9.8%)	3 (8.3%)	0.258
MAS as first manifestation	43 (36.4%)	24 (29.3%)	19 (52.8%)	0.015
Splenomegaly	102 (86.4%)	68 (82.9%)	34 (94.4%)	0.092
Concomitant infections	46 (39.0%)	33 (40.2%)	13 (36.1%)	0.672
Laboratory parameters, n (%) or median (range)				
WBC, ×10^9^/L	5.20 (0.17, 34.83)	5.08 (0.17, 34.83)	5.72 (0.50, 29.30)	0.628
Neu, ×10^9^/L	3.38 (0.02, 30.23)	3.24 (0.02, 30.23)	4.07 (0.13, 27.10)	0.511
Hgb, g/L	94.00 (48.00, 196.00)	94.00 (48.00, 196.00)	95.50 (60.00, 153.00)	0.659
PLT, ×10^9^/L	93.50 (5.00, 471.00)	84.50 (5.00, 382.00)	119.00 (13.00, 471.00)	0.124
ALT, U/L	65.95 (2.10, 3251.00)	95.90 (2.10, 2252.00)	50.00 (5.00, 3251.00)	0.213
AST, U/L	84.00 (7.65, 3250.70)	98.50 (7.65, 3250.70)	50.75 (12.00, 1757.00)	0.289
TG, mmol/L	2.99 (0.94, 10.20)	3.12 (0.94, 10.20)	2.80 (1.00, 4.39)	0.290
Fbg, g/L	1.80 (0.40, 11.18)	1.74 (0.56, 11.18)	1.85 (0.40, 5.81)	0.492
SF, µg/L	2277.00 (40.10, 112954.00)	2000.00 (466.00, 100000.00)	3423.15 (40.10, 112954.00)	0.831
sCD25, pg/mL	11208.50 (639.00, 94444.00)	9875.15 (639.00, 94444.00)	11812.60 (2337.00, 26732.00)	0.840
NK cell activity, %	13.57 (0.51, 22.30)	13.39 (0.51, 19.12)	13.69 (1.10, 22.30)	0.618
Hemophagocytosis phenomenon in bone morrow, n	70 (59.3%)	47 (57.3%)	23 (63.9%)	0.503

^a^Patients whose initial therapy is the glucocorticoid-based regimen.

^b^Patients whose initial therapy is the etoposide-based regimen.

AOSD, adult-onset Still’s disease; SLE, systemic lupus erythematous; UCTD, undifferentiated connective tissue disease; RA, rheumatoid arthritis; DM, dermatomyositis; SS, Sjogren syndrome; AIH, autoimmune hepatitis; BD, Bechet’s disease; WBC, white blood cell count; Neu, neutrophil count; HGB, hemoglobin concentration; PLT, platelet count; ALT, alanine aminotransferase; AST, aspartate aminotransferase; T-BIL, serum total bilirubin; I-BIL serum indirect bilirubin; D-BIL serum direct bilirubin; TG, triglycerides; Fbg, fibrinogen; Fer, serum ferritin; sCD25, soluble CD25.

### Therapies and outcomes of patients

Patients were divided into two groups according to the initial treatment. Group 1 (n = 82) patients initially received glucocorticoid-based regimen. Group 2 (n = 36) patients initially received etoposide-based regimen. Comparing the baseline laboratory data between two groups, no significant difference was noted.

Two weeks after initial treatment, the overall response (OR), CR, and PR rates in group 1 was 37.8% (31/82), 7.3% (6/82), and 30.5% (25/82), respectively. Of 51 patients who did not achieve remission, six continued glucocorticoid-based treatment, of whom two (2/6, 33.3%) achieved PR and four (4/6, 66.7%) died of uncontrollable MAS. Forty-five patients switched over etoposide-based treatment. Two weeks after altering the treatment regimen, 38 patients (38/45, 84.4%) achieved remission, with a CR rate of 6.7% (3/45) and a PR rate of 77.8% (35/45). Four patients (4/45, 8.9%) remained with NR, and three (3/45, 6.7%) died. Four weeks after altering the treatment, the rates of CR, PR, and NR were 20.0% (9/45), 66.7% (30/45), and 4.4% (2/45), respectively, and the death toll increased to 4 (4/45, 8.9%). In the subsequent treatment, those two NR patients reached remission. The OR rate of switching to a VP-16-based treatment was significantly higher than continuing glucocorticoid-based treatment in refractory patients (84.4% *vs*. 33.3%, P = 0.020).

**Figure 1:**
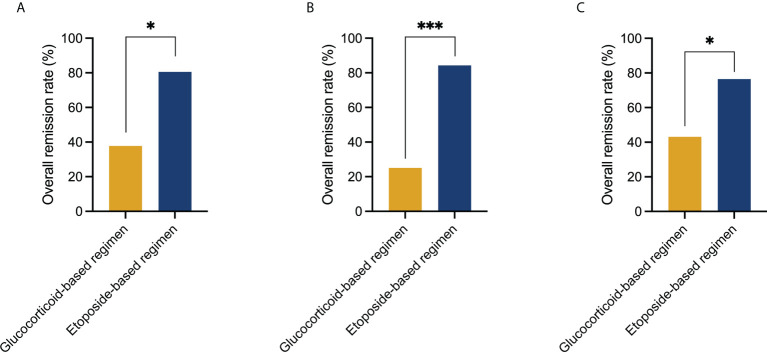
Comparison of treatment response to the glucocorticoid-based regimen and etoposide-based regimen at 2 weeks post initial treatment. **(A)** Initial use of the etoposide-based regimen induced a higher overall remission rate than the glucocorticoid-based regimen in the entire cohort (80.6% *vs*. 37.8%, P = 0.020). **(B)** Initial use of the etoposide-based regimen induced a higher overall remission rate than the glucocorticoid-based regimen in patients who had MAS as first manifestation (84.2% *vs*. 25%, P < 0.001). **(C)** Initial use of the etoposide-based regimen induced a higher overall remission rate than the glucocorticoid-based regimen in patients who had MAS after diagnosis of autoimmune disease (76.5% *vs*. 43.1%, P = 0.016). Statistically significant differences between each patient group are shown as *P < 0.05, ***P < 0.001.

For group 2, the OR, CR, PR, and NR rates at the 2-week post initial treatment were 80.6% (29/36), 22.2% (8/36), 58.3% (21/36), and 19.4% (7/36), respectively. At 4 weeks, the OR, CR, and PR rates were 88.9% (32/36), 27.8% (10/36), and 61.1% (22/36), respectively. Four patients (4/36, 11.1%) remained with NR, of which one obtained PR at 6 weeks and three died. The 2-week posttreatment OR and CR rates in group 2 were significantly higher compared with group 1 (**Figure 1**).

Twenty patients experienced MAS recurrence during the follow-up. Twelve cases (12/74, 16.2%) were reported from group 1 and eight (8/33, 24.2%) from group 2. There was no statistical difference in the incidence of relapse between the two groups (P = 0.325). At relapse, one patient received glucocorticoid-based treatment and obtained PR. Nineteen patients received the VP-16-based regimen, 18 (18/19, 94.7%) reached remission, with a CR rate of 31.6% (6/19) and a PR rate of 68.4% (13/19), and one patient died of uncontrollable MAS. Two patients (2/106, 1.9%) had a second MAS recurrence, one achieved remission after etoposide contained regimen, and one died due to MAS progression.

### Occurrence of MAS in different stages of AID

In 43 patients (43/118, 36.4%), HLH occurred as the first of an occult AID. Seventy-five patients (75/118, 63.6%) had MAS after the diagnosis of AID, of whom the diagnostic interval from AID to MAS was 2 (0.5–359) months. Concomitant infections were more common in the latter cohort, with a P value closely approaching statistical significance (12/45, 27.9% *vs*. 33/75, 44.0%, P = 0.083). While the incidence of MAS recurrence is similar (10/43, 23.3% *vs*. 10/75, 13.3%, P = 0.167). The initial use of the etoposide-based regimen reached a higher OR rate in both cohorts (P < 0.05) (**Figure 1**).

### Adverse reactions of the etoposide-based regimen

#### Infection

A total of 92 patients received the VP-16-based treatment in this study. Before the etoposide-based regimen, 49 (49/92, 53.3%) patients had radiological signs of pulmonary infection. Six patients (6/92, 6.5%) had infections confirmed by positive cultures, including *Klebsiella pneumoniae*, *Acinetobacter baumannii*, *Pseudomonas aeruginosa*, and *Candida albicans*. During the course of treatment, 21 patients (21/92, 22.8%) developed exacerbated infections, and 12 patients (12/92, 13.0%) presented with new infections. With appropriate antibiotic treatment, symptoms of infections improved in most patients. Four patients (4/92, 4.3%) died of unremitting MAS with severe infections.

#### Bone marrow suppression and cytopenia

Bone marrow biopsy was repeated in 3~4 weeks after commencing the etoposide-based regimen in 86 patients. No evidence of bone marrow suppression was observed. The Hgb [101.00(48.00, 142.00)g/l *vs*. 87.00(36.00, 153.00)g/l, P = 0.005] and PLT [186.00(7.00, 452.00) × 10^9^/l *vs*. 105.00(13.00, 707.00) × 10^9^/l, P < 0.001] levels of patients were improved compared with baseline while no significant difference was noted in the level of WBC [6.15(0.15, 47.34) × 10^9^/L *vs*. 5.46(0.50, 49.17) × 10^9^/l, P = 0.241] and Neu [4.55(0.00, 33.45) × 10^9^/l *vs*. 3.49(0.03, 41.87) × 10^9^/l, P = 0.217].

### Comparison of serum cytokine levels in different MAS stages

**Figure 2:**
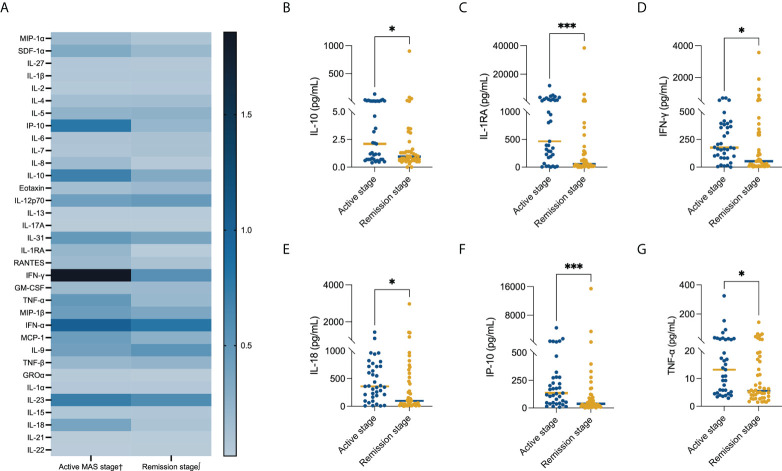
Comparison of the serum cytokine level in different MAS stages. Serum levels of **(A)** all evaluated cytokines, **(B)** IL-1 (P = 0.041), **(C)** IL-1RA (P < 0.001), **(D)** IFN-γ (P = 0.013), **(E)** IL-18 (P = 0.021), **(F)** IP-10 (P = 0.001), and **(G)** TNF-α (P = 0.020). The bars represent median values. Statistically significant differences between each patient group are shown as *P < 0.05, ***P < 0.001. †Data available in 37cases. ∫Data available in 46 cases.

**Figure 3:**
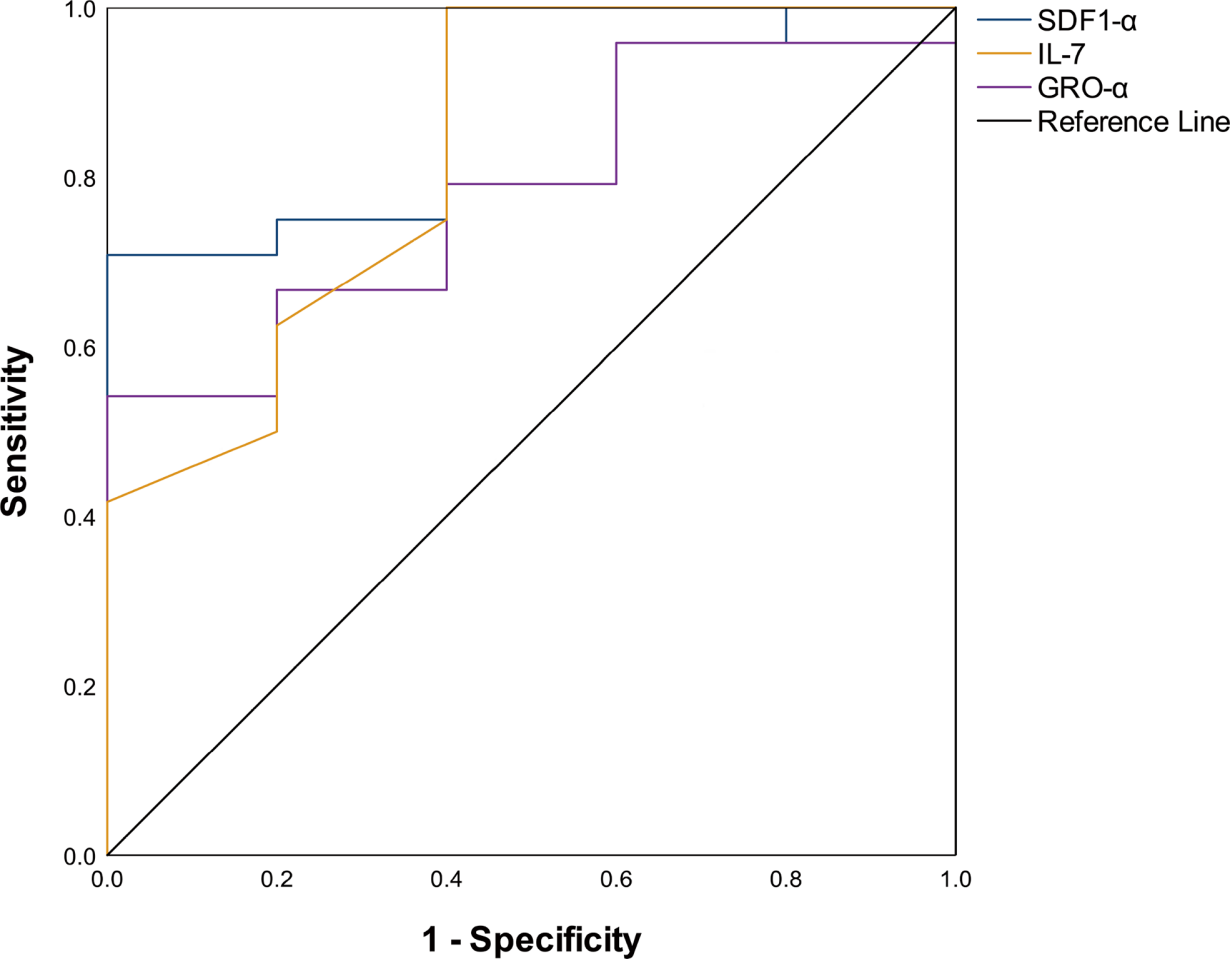
ROC curve for cytokines.

Comparing the serum cytokine profiles in the active stage and remission stage, significant differences were noted in IP-10 [134.80(8.00, 4475.00) pg/ml *vs*. 37.45(1.20, 15,423.00) pg/ml, P = 0.001], IL-10 [2.10(0.40, 133.50) pg/ml *vs*. 0.95 (0.00, 904.70) pg/mL, P = 0.041], IL-1RA [466.00(0.00, 12,013.00) pg/ml *vs*. 58.46(0.00, 38,504.00) pg/ml, P < 0.001], IFN-γ [176.42(0.00, 701.35) pg/ml *vs*. 53.50(3.33, 3,577.00) pg/ml, P = 0.013], TNF-α [13.20(2.90, 324.44) pg/ml *vs*. 5.55(1.48, 143.70) pg/ml, P = 0.020], and IL-18 [359.10(6.50, 1,438.00) pg/ml *vs*. 96.80(0.62, 2,967.00) pg/ml, P = 0.021] levels (**Figure 2**). We compared patients who were refractory to the glucocorticoid-based regimen to those who were not. The results illustrated that the levels of SDF-1α (P = 0.018) and IL-7 (P = 0.022) were higher in refractory patients, while the GRO-α level had a strong tendency toward statistical significance (P = 0.050) (**Table 2**). The ROC curve was employed to further evaluate the diagnostic ability of SDF-1α, IL-7, and GRO-α (**Figure 3**).

**Table 2:** Comparison of serum SDF-1α, IL-7, and GRO-α in refractory patients and regular patients.

Parameters	Refractory group	Regular group	P value	Cutoff value	AUC	Sensitivity	Specificity
SDF-1α (pg/mL)	647.10 (58.20-2291.00)	333.80 (32.89-503.80)	0.018	510.30	0.842	70.8%	100%
IL-7 (pg/mL)	0.80 90.30-25.40)	0.29 (0.20-1.00)	0.022	0.32	0.829	87.5%	60%
GRO-α (pg/mL)	17.54 (1.00-91.80)	1.70 (1.18-11.00)	0.050	13.25	0.783	54.2%	100%

SDF-1α, stromal cell-derived factor 1 alpha; ROC, receiver-operating characteristic curve; AUC, area under the curve. The optimal cutoff value was obtained by calculating the maximum Youden index (sensitivity + specificity – 1). AUC is based on the area under the ROC curve shown in **Figure 3**.

**Figure 4:**
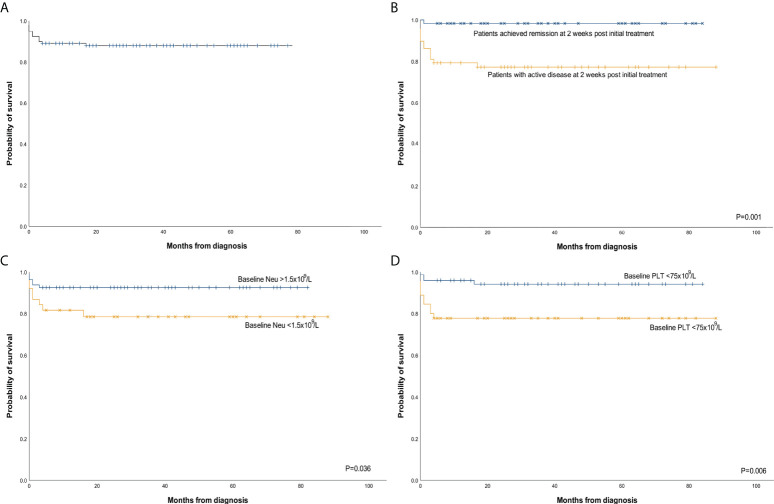
Kaplan–Meier survival curves. **(A)** Overall survival (OS) for the entire cohort (n = 118). **(B)** OS by the MAS status at the 2-week post initial treatment (P = 0.001). **(C)** OS by the baseline Neu level (P = 0.036). **(D)** OS by the baseline PLT level (P = 0.006). MAS, macrophage activation syndrome; Neu, neutrophil count; PLT, platelet count.

### Overall survival and prognostic factors

At a median follow-up of 31 (1–88) months, the 3-month, 6-month, and 3-year probability of survival was 89.8% (95% confidence interval [CI]: 84.3–95.3), 89.0% (95% CI: 83.3–94.7), and 87.9% (95% CI: 82.0–93.8), respectively (**Figure 4A**). The overall mortality was 11.9% (14/118), and 12 patients (12/14, 85.7%) died within 3 months after MAS diagnosis. Thirteen patients (13/118, 11.0%) died of MAS progression, the specific causes including severe infectious (7/118, 5.9%), hemorrhage (4/118, 3.4%), central nervous system involvement (1/118, 0.8%), and multiple-organ failure (1/118, 0.8%). One patient (1/118, 0.8%) died of coagulation dysfunction caused by SLE at the remission stage of MAS.

In univariate analysis, the active MAS status at the 2-week post initial treatment (P = 0.001) (**Figure 4B**), baseline Neu <1.5 × 10^9^/l (P = 0.036) (**Figure 4C**), and baseline PLT <75 × 10^9^/l (P = 0.006) (**Figure 4D**) were associated with inferior survival. The other tested factors such as age (P = 0.052), gender (P = 0.216), underlying AID (P = 0.320), hepatomegaly (P = 0.783), splenomegaly (P = 0.445), hemophagocytosis in bone marrow (P = 0.667), or HLH as the first manifestation (P = 0.620) were not significantly associated with OS in univariate analysis. In 107 evaluable patients, MAS recurrence was associated with inferior survival (P = 0.042). In multivariate analysis, the prognostic influence of MAS status at the 2-week post initial treatment, baseline Neu <1.5 × 10^9^/l, and baseline PLT <75 × 10^9^/l were analyzed. Data suggest that active MAS status at the 2-week post initial treatment [P = 0.009, HR = 15.281, 95% CI, (0.1.972, 118.430)] and baseline Neu <1.5 × 10^9^/l [P = 0.017, HR = 3.678, 95% CI (1.267, 10.672)] were independent risk factors for the prognosis of MAS patients while baseline PLT <75 × 10^9^/l was on the boundary of significance [P = 0.057, HR = 3.101, 95%CI (0.968, 9.934)].

## Discussion

HLH is a rapidly progressive disease with dismal prognosis; the median survival was only 1~2 months if appropriate treatment was not initiated promptly (13). Early diagnosis and appropriate treatment are the keys to improving the outcomes. However, early diagnosis remains a challenge. MAS can occur in all stages of AID or as the first manifestation. Cardinal symptoms of MAS overlap with active AID or AID combined with infection, which could lower the vigilance of physicians. Cytopenia is an easily accessible alarming indicator of HLH, but in MAS, initial blood changes may be less pronounced. Soluble CD25 and NK cell activities are valuable parameters for early diagnosis but have not been widely rolled out (14, 15). Thus, it is hard to identify MAS at the early stage, consequently resulting in delayed treatment. In our study, we noticed that MAS appears to be more prevalent within 2 months of AID onset. Less than 20% of patients in our study displayed neutropenia, and the incidence of anemia was relatively low (16). The descending trend of blood cells might be more indicative (17, 18). In a recent study, it is proved that PLT <100 × 10^9^/l, SF >6,000 ug/l and the evidence of enlargement of the liver and spleen are the predictive factors of MAS (19). Briefly speaking, we are of the opinion that for newly diagnosed AID patients, MAS should be considered if patients develop unremitting fever, descending blood cell counts, or elevated SF levels, especially for AOSD patients.

The HLH treatment strategy consists of two parts: the short-term aim is to suppress the severe hyperinflammatory to reduce the early mortality. The long-term aim is to cure the underlying disease to achieve long-term survival (6, 13). The long-term survival of AID patients is favorable, which leaves the control of HLH as the key to improving the outcomes (9, 11, 20, 21). To date, there is no validated management guideline for adult MAS due to the lack of random randomized control trials. Based on pediatric experience, glucocorticoid pulse therapy is conventionally the first-line treatment. In a single-center retrospective study, 19 patients were treated with glucocorticoid therapy; all patients had a good outcome without any mortality (22). However, in the study by Fukaya et al, 54% of patients did not respond to high-dose glucocorticoid (8). In a large cohort of 89 adult patients, the response rate to glucocorticoid therapy was 63% (23). Unfortunately, the OR rate of the glucocorticoid-based regimen is unsatisfactory in our study, which looks slightly lower than previous data (8, 23). A lot of patients were referred to our center, and that might increase the proportion of refractory patients in our cohort, leading to a lower OR to the glucocorticoid-based regimen. Management of refractory patients lacks formal standards. However, cytokine-targeted treatment has become an attractive alternative with the increased knowledge of hypercytokinemia in MAS (24).

Anakinra, a recombinant IL-1 receptor antagonist, is one of the most compelling biological agents in the treatment of MAS. A remarkable improvement in response to anakinra after an inadequate response to glucocorticoid has been reported (25–28). In our study, the serum levels of IL-1RA, a natural occurring antagonist of IL-1, were significantly decreased in the remission phase compared to the active phase. Serum level of another anti-inflammatory factor (29), IL-10, was also decreased in the remission phase. These findings indicate that anti-inflammatory factors are elevated to counteract the excessive inflammation in the pathogenesis of MAS. It is well recognized that overproduction of IFN-γ plays a pivotal role in the pathogenesis of HLH (30–32). In the murine MAS model, the use of the IFN-γ monoclonal antibody improved the survival (31). In a patient who was unresponsive to high-dose glucocorticoid and anakinra, emapalumab treatment achieved prompt and sustained response (33). However, the efficacy of the IFN-γ monoclonal antibody in MAS remains to be further investigated. Our data show that serum levels of IFN-γ, IL-18, and IP-10 decreased significantly at the remission stage. IL-18 is the inducer of IFN-γ, while IP-10 is induced by IFN-γ. These findings suggest the important role of the IFN-γ pathway in the pathogenesis of MAS, providing the theoretical basis for the application of the IFN-γ monoclonal antibody.

In addition to biological agents, the etoposide-based regimen is also an option for refractory patients (12, 34), especially for patients who have no access to biologics. In the study by Horne et al., administrating etoposide in rapidly progressing patients achieved satisfactory results. All children responded very well to etoposide-contained therapy (35). In the study by Wang et al., the use of etoposide achieved a response rate of 80% (34). However, Naymagon et al. concluded that there was no significant difference in survival between the etoposide and no-etoposide groups (36). In a system review, patients with Kawasaki disease and MAS who were treated with HLH protocol presented higher mortality (37). However, the comparison of baseline data was lacking in this study. Furthermore, the sample size of the above studies was relatively small. Here in this large cohort, we further investigated the efficacy of the etoposide-based regimen in adult MAS. In this study, the OR rate of refractory patients to the etoposide-based regimen was as high as 84.4%. Due to the retrospective nature of this study, the choice of initial treatment, although deliberated, was not able to follow rigorous criteria. The selection of initial treatment is based primarily on the severity of the condition and whether the underlying cause is clear. A small portion of patients was initially treated with the etoposide-based regimen and achieved a significantly higher 2-week OR rate than patients who were initially treated with the glucocorticoid-based regimen. For patients with relapsed MAS, the OR to etoposide-based regimen was also satisfactory. The risk of infection and bone marrow suppression carried by the etoposide-based regimen is what concerns physicians. However, it should be emphasized that if the active MAS sustained, the processive immune dysfunction will make the infection hard to control. The severe anemia and thrombocytopenia induced by active MAS could also endanger patients’ lives. Our results showed that the incidence of new infection or exacerbated infection was acceptable. Infections were controllable under appropriate anti-infection treatment in most cases. Moreover, we noticed that at diagnosis, infections were more common in patients who developed MAS after AID, presumably owing to the former treatment for AID. Intensive anti-infection therapy might be necessary for these patients. Bone marrow toxicity related to the etoposide-based regimen was not observed. Four weeks after etoposide-based treatment, the PLT and Hgb levels of patients were remarkably improved. No evidence of secondary malignancy was observed in our study. In a recent study, it is proved that moderately dosed etoposide may be beneficial in severe and/or refractory MAS-HLH. The dose reduction of etoposide might lead to a lower rate of side effects but is sufficient for MAS treatment, which might be a new direction for future treatment (35). Regardless, our data demonstrated that the etoposide-based regimen is effective and tolerable for adult MAS, whether in initial treatment, in refractory, or in recurrence.

The overall prognosis is favorable in this study, with a mortality of 11.9% as compared to 41% in sHLH (38). Most patients died within 3 months after MAS onset. Closer monitoring of the MAS condition and intensive support treatment at the early stage after diagnosis might contribute to better outcomes. Our results showed that baseline Neu <1.5 × 10^9^/l [P = 0.017, HR = 3.678, 95% CI (1.267, 10.672)] is an independent risk factor for the prognosis of MAS patients, while baseline PLT 75 × 10^9^/l was on the boundary of significance [P = 0.057, HR = 3.101, 95% CI (0.968, 9.934)]. Lower baseline Neu and PLT counts might indicate a delayed diagnosis or severe condition as AID typically results in elevated Neu and PLT. To improve baseline blood cell counts, an early diagnosis is essential. An active MAS status at the 2-week post initial treatment is also an independent risk factor for the prognosis. Therefore, selecting an effective treatment to obtain remission as early as possible is warranted. Our results showed that the etoposide-based regimen achieved a high OR rate at 2 weeks post initial treatment, but most patients could still benefit from the etoposide-based regimen after the refractory to glucocorticoid-based regimen. It is hard to balance the risk of delayed remission with the risk of adverse reactions when selecting the initial treatment. Thus, it is critical to identify refractory patients in the early stage. Our obtained results revealed that baseline SDF-1α, IL-7, and GRO-α levels were comparatively higher in refractory patients. Further analysis suggested that SDF-1α, IL-7, and GRO-α are the cytokines that predict refractory. It is reported that SDF-1α significantly increased growth factor-independent proliferation of colony-forming unit granulocyte-macrophage induced by ITD-Flt3 (39). GRO-α is one of the ligands of CXCR2, upon binding to the receptor, which induces recruitment of macrophages (40). High levels of SDF-1α and GRO-α might indicate stronger overactivation of macrophages. IL-7 has been found to stimulate the growth of T cells (41). In a recent study, it is proved that exogenous IL-7 can be beneficial in overcoming radiation-induced lymphopenia (42). It is possible that IL-7 can also be beneficial in overcoming glucocorticoid or chemotherapy-induced lymphopenia, resulting in refractory MAS. For those patients, early use of etoposide might improve the outcomes.

In univariate analysis, MAS recurrence is associated with inferior survival. This is no surprise given the re-impairment induced by the inflammatory factors and cells. We have sought to identify risk factors for recurrence but to no avail (data not shown). However, uncontrollable MAS is still the major death cause in relapsed patients. Timely administrating a proper treatment is important.

## Conclusion

We concluded that MAS typically occurs within 2 months after the onset of autoimmune disease in adults. SDF-1α, IL-7, and GRO-α could be used to predict refractory MAS. The etoposide-based regimen is effective and tolerable for adult MAS, whether in initial treatment, in refractory, or in recurrence.

## Data availability statement

The raw data supporting the conclusions of this article will be made available by the authors, without undue reservation.

## Ethics statement

This study was reviewed and approved by Ethics Committee of Beijing Friendship Hospital, Capital Medical University. Written informed consent for participation was not required for this study in accordance with the national legislation and the institutional requirements.

## Author contributions

LH, YW designed the study and analyzed data. LH wrote the paper. LH, SY, RZ performed data acquisition. LH, SY, ML, ZH, ZW, and HZ performed the statistical analysis. All authors contributed to the article and approved the submitted version.

## Funding

This work was supported by the National Natural Science Foundation of China [82170122 and 81871633].

## Conflict of interest

The authors declare that the research was conducted in the absence of any commercial or financial relationships that could be construed as a potential conflict of interest.

## Publisher’s note

All claims expressed in this article are solely those of the authors and do not necessarily represent those of their affiliated organizations, or those of the publisher, the editors and the reviewers. Any product that may be evaluated in this article, or claim that may be made by its manufacturer, is not guaranteed or endorsed by the publisher.
